# Taxonomically-linked growth phenotypes during arsenic stress among arsenic resistant bacteria isolated from soils overlying the Centralia coal seam fire

**DOI:** 10.1371/journal.pone.0191893

**Published:** 2018-01-25

**Authors:** Taylor K. Dunivin, Justine Miller, Ashley Shade

**Affiliations:** 1 Department of Microbiology and Molecular Genetics, Michigan State University, East Lansing, Michigan, United States of America; 2 Environmental and Integrative Toxicological Sciences Doctoral Program, Michigan State University, East Lansing, Michigan, United States of America; 3 Lyman Briggs College, Michigan State University, East Lansing, Michigan, United States of America; 4 Program in Ecology, Evolutionary Biology and Behavior, Michigan State University, East Lansing, Michigan, United States of America; University of Illinois at Urbana-Champaign, UNITED STATES

## Abstract

Arsenic (As), a toxic element, has impacted life since early Earth. Thus, microorganisms have evolved many As resistance and tolerance mechanisms to improve their survival outcomes given As exposure. We isolated As resistant bacteria from Centralia, PA, the site of an underground coal seam fire that has been burning since 1962. From a 57.4°C soil collected from a vent above the fire, we isolated 25 unique aerobic As resistant bacterial strains spanning seven genera. We examined their diversity, resistance gene content, transformation abilities, inhibitory concentrations, and growth phenotypes. Although As concentrations were low at the time of soil collection (2.58 ppm), isolates had high minimum inhibitory concentrations (MICs) of arsenate and arsenite (>300 mM and 20 mM respectively), and most isolates were capable of arsenate reduction. We screened isolates (PCR and sequencing) using 12 published primer sets for six As resistance genes (AsRGs). Genes encoding arsenate reductase (*arsC*) and arsenite efflux pumps (*arsB*, *ACR3(2)*) were present, and phylogenetic incongruence between 16S rRNA genes and AsRGs provided evidence for horizontal gene transfer. A detailed investigation of differences in isolate growth phenotypes across As concentrations (lag time to exponential growth, maximum growth rate, and maximum OD_590_) showed a relationship with taxonomy, providing information that could help to predict an isolate’s performance given As exposure *in situ*. Our results suggest that microbiological management and remediation of environmental As could be informed by taxonomically-linked As tolerance, potential for resistance gene transferability, and the rare biosphere.

## Introduction

Arsenic (As), a toxic metalloid, is naturally present in soil, but levels are generally low (<10 ppm) [1]. Because of the ubiquity of As and its toxicity, bacteria have evolved a variety of As-specific detoxification mechanisms [2]. Bacterial strains have been shown to oxidize, reduce, methylate, and demethylate As [3]. The toxicity and mobility of As can change depending on its oxidation state with arsenate (As^5+^) being less soluble and less toxic than arsenite (As^3+^) [4]; thus, environmental bacteria are considered important constituents of the biogeochemical cycling of As because the presence and transfer of the resistance genes encoding these activities affect the mobility of As.

As resistance genes (AsRGs) can be located on chromosomes, plasmids, or both [2]. Several studies indicate that horizontal gene transfer (HGT) has occurred with AsRGs [5–9], suggesting the potential exists for AsRGs to propagate in a microbial community given a selective pressure of As exposure; however, timing of HGT is difficult to determine [9]. In addition to As-specific mechanisms of resistance conferred by AsRGs, microorganisms can also employ nonspecific and transient cellular mechanisms to withstand As exposure. Cell envelope permeability to As, oxidative stress response, and heat shock proteins have all been shown to be differentially regulated in response to As [2,10–14]. These are collectively referred to as As tolerance mechanisms [11,15]. However, tolerance in the absence of resistance (i.e. AsRGs) is often not enough to enable cell survival given lasting As exposure [15].

Much of the current understanding of As resistance and tolerance has come from the detailed study of As resistant isolates that have been cultivated from As contaminated sites (e.g., [5,6,16–21]. More broadly, culture-dependent approaches to improve knowledge of microbial diversity and functions are experiencing a renaissance in today’s age of high-throughput meta ‘omics (e.g., [22–24]. In addition to direct assessment of physiology and functional capabilities, characterized isolates can provide high quality genome references for culture-independent metagenome and single-cell genome assemblies [25–27]. Thus, culture-dependent approaches continue to offer opportunity to examine several aspects of As resistance that are not captured with culture-independent approaches. For example, growth phenotypes in As and minimum inhibitory concentrations (MICs) are best determined directly with isolates. Additionally, it is difficult to assess potential horizontal gene transfer (HGT) from culture-independent methods [28,29], and HGT is an important consideration in AsRG ecology. Finally, cultured isolates provide access to microorganisms that may be used to support applications like bioremediation of contaminated sites (e.g., [25,27]). Though isolate collections do not provide comprehensive knowledge of microbial diversity and are limited by cultivation conditions, these collections can be used to inform isolate ecology in the context of their larger microbial community, especially when coupled with culture-independent approaches (e.g., [30]).

The underground coal seam fire in Centralia, PA ignited in 1962 and has been burning ever since. The soil microbial communities overlying the underground fire experience a multitude of fire-related stressors, including high temperatures and exposure to coal combustion products and CO, CO_2_, and NH_4_ gas emissions; these coal fire pollutants impact local biogeochemistry [31–33]. Because As is naturally present in coal, exposure to the coal seam fire is expected to influence soil microbial As resistance and AsRG transfer. Along with lead, zinc, mercury, and copper, As has been documented in increased concentrations near active vents, which are steaming surface fissures created by instability from the underground coal fire [34].

Our objective was to characterize As resistant bacterial isolates from an active thermal vent (57.4°C) in Centralia in order to expand knowledge of the characteristics of As resistant bacterial isolates from a coal mine contaminated site. This knowledge will improve metagenome analysis and genomic analysis of similar organisms, as there is a move towards expanding culture collections and knowledge of cultivated organisms (e.g., [27]). We aimed to gain insights into their genetic mechanisms of As resistance, growth consequences under increasing arsenite and arsenate exposure, and potential for interspecies transfer of As resistance. Our culture-dependent approach provided insights into isolate distinctions in growth phenotypes given As exposure. Considering culture-independent information (16S rRNA gene amplicon sequencing) additionally allowed us to determine the relative contributions of these isolates to their larger community. These findings bring to light complexities of predicting microbial community-level response to As.

## Materials and methods

### Soil collection and site description

The Pennsylvania Department of Environmental Protection provided permission to access to the field site. The field site is not a protected area. This work did not involve endangered or protected species. This study did not involve vertebrates. A soil surface core (20 cm depth and 5.1 cm diameter) was collected in October 2014 from an active vent (steam escaping) in Centralia, Pennsylvania. This vent (site Cen13, GPS coordinates: 40 48.070, 076 20.574) was selected because it has had historical fire activity since at least 2007 [33] and was the hottest detected at the time of sampling with a measured surface temperature (10 cm depth) of 57.4°C (ambient air temperature was 13.3°C). Detailed soil geochemical data was assayed by the Michigan State University Soil and Plant Nutrient Laboratory (East Lansing, MI, USA, http://www.spnl.msu.edu/) according to their standard protocols, and total As was measured by Element Materials Technology using the Environmental Protection Agency’s method 3050B for sample preparation and ICP-MS (S1 Table). Upon sampling, the soil was kept on ice until transport to the lab where it was manually homogenized, sieved through 4 mm mesh, and stored at -80°C until further processing.

### Cultivation-dependent soil bacterial community growth

Five grams of soil was removed from -80°C and kept at 4°C for 48 h. The soil was warmed to room temperature for 1 h and then suspended in 25 mL of sterile Dulbecco’s phosphate-buffered saline (ThermoFisher; dPBS), vortexed for 2 min, and allowed to settle for 2 min. The supernatant was plated onto 50% tryptic soy agar (Becton Dickinson and Company; TSA50) with 200 μg mL^-1^ of cycloheximide added to inhibit fungal growth. Plates were incubated at 27°C for 24 h. To obtain a culture-dependent bacterial community representative of these growth conditions, overgrown plates were scraped to make a 25% glycerol stock and stored at -80°C for future assays.

### Isolation of As resistant bacteria

Twenty mL of trypticase soy broth (TSB50) was inoculated with the bacterial community glycerol stock and grown for 6 h with shaking at 200 rpm and 12 mm amplitude. As was not included in the medium to avoid transfer of AsRGs. The culture was plated onto TSA50 with either 10 mM Na_2_HAsO_4_ or 1 mM NaAsO_2_ to screen for arsenate or arsenite resistant colonies, respectively. Ninety-four total colonies (35 from sodium arsenate; 59 from sodium arsenite) were streaked to purity (3x) on their respective media type; 69 pure isolates were recovered and made into 25% glycerol stocks for long term storage at -80°C. From these pure cultures, 25 distinct isolates were identified by genotype with 16S rRNA gene sequencing and by phenotype using MIC assays.

### Morphological characterization and temperature maxima

Overnight cultures of isolates grown in 3 mL TSB50 were examined using a Nikon E800 Eclipse microscope. Cell morphology was visualized using a photometrics CoolSnap MYO microscope camera (Tuscan, AZ, USA) and Micromanager 4.22 [35] was used for image acquisition. Cell size was measured using Fiji image analysis software [36]. Colony morphology on TSA50 plates was imaged after incubation at 27°C for 24 h. To measure growth temperature maxima, isolates (2% culture in fresh TSB50) were incubated in a T100 Thermo Cycler (BioRad) for 24 h with a thermal gradient (32–52°C). Optical density at 590 nm (OD_590_) was measured using an Infinite F500 plate reader (Tecan). The maximum temperature for growth was determined as the highest temperature with an increase in OD_590_ from background. This process was repeated for a minimum of two biological replicates per isolate.

### DNA extraction and quantification

Freezer stocks of isolates were inoculated into 3 mL TSB50 and shaken at 27°C at 200 rpm with a 12 mm amplitude until turbid. Genomic DNA (gDNA) was extracted using the E.Z.N.A. Bacterial DNA Kit (Omega Bio-Tek) according to the manufacturer’s instructions. Isolated gDNA was quantified with fluorometry using the Qubit dsDNA broad range assay kit (Invitrogen) and a Qubit 2.0 (Invitrogen) according to the manufacturer’s instructions. DNA was stored in sterile Tris-EDTA buffer (Sigma; pH 8) at -20°C.

### Endpoint PCR and amplicon sequencing

The near full length 16S rRNA gene was amplified for each isolate using the universal primer pairs Uni-27F and Uni-1492R (S2 Table). PCR amplification of 16S rRNA was carried out in a T100 Thermo Cycler (BioRad) using 25 μL total volume including 30 ng genomic DNA, 0.4 μM of each primer, 0.8 mM dNTPs (Sigma), 2.5 μL 10X Pfu Buffer (Promega), 2X high fidelity Pfu DNA Polymerase (Promega), and nuclease free water to a final volume of 25 μL. The 16S rRNA PCR reaction cycle included a 2 min initial denaturation at 95°C, 30 cycles of denaturation at 95°C for 30 s, annealing at 55°C for 30 s, extension at 72°C for 1 min, and a final extension at 72°C for 10 min. PCR products were run on a 1% agarose gel for 45 min at 700 mV. The PCR product of 1.4 kb from the 16S rRNA gene was gel extracted using the Wizard SV Gel and PCR Clean Up System (Promega) according to the manufacturer’s instructions. Gel extraction products were quantified as described above. Purified 16S rRNA amplicons were sequenced using the ABI Prism BigDye Terminator Version 3.1 Cycle sequencing kit by the Michigan State University Genomics Core Research Technology Support Facility. Forward and reverse 16S rRNA sequences were aligned using CAP3 (v. 3.0,[37]) to obtain near full length 16S rRNA gene sequences, except for isolates A2707, A2723, and A2735 which could not be sequenced using the 1492R primers. For these three isolates, primer U515F [38] was used to obtain a near-full length 16S rRNA sequence. Sequences were assigned taxonomy using both the Ribosomal Database Project (RDP) 16S rRNA database (v. 2.10, [39]) and the EzTaxon server [40].

Isolates were screened for the following AsRGs: *arsB*, *ACR3(1)*, *ACR3(2)*, *arsC*, *arrA*, *aioA*, and *arsM* using published primers that were chosen because of their continued use in the literature (S2 Table; [5,7,41–44]). All PCRs were carried out with published reaction conditions in a T100 Thermo Cycler (BioRad). While amplicons were obtained for all primer sets used, only products confirmed by sequencing were considered positive hits. Once a product was confirmed, the PCR was repeated using the confirmed isolate as a positive control. All amplicons were gel extracted and sequenced as described above. At least one forward and one reverse gene sequence was merged in CodonCodeAligner (v. 6.0.2, Codon Code Corporation) to create AsRG contigs. All sequences >200 bp were submitted to NCBI, and sequences can be accessed from GenBank with the following accession numbers: 16S rRNA KX825887- KX825911, *arsC* KY405022- KY405029, *ACR3(2)* KY405030- KY405032, and *arsB* KY405033- KY405040. Four *arsC* contigs were < 200 bp and are included in S3 Table. Amino acid sequences for each protein-coding gene are also available in NCBI GenBank.

### Phylogenetic analysis

To compare the 16S rRNA phylogenetic diversity of Centralia As resistant isolates to previous reports, isolates from existing literature were included in the phylogenetic analysis. Only studies with both 16S rRNA sequences > 700 bps and confirmed As resistance (selection on As-containing media) were included. Ultimately 6 studies [5,18,45–48] were included, and all sequences from relevant lineages were included in the final tree (55 sequences total). Closest 16S rRNA gene relatives deposited at the NCBI (http://www.ncbi.nlm.nih.gov/) were also included in the analysis. Sequences were aligned using the RDP aligner [49]. RDP characters were removed from aligned sequences using BioEdit (v. 7.2.5, [50]). 16S rRNA gene trees were made with MEGA7.0 [51] and constructed with the Neighbor-joining algorithm using the Kimura 2 parameter model with 1000 bootstrap replications.

To examine the phylogeny of *arsC*, *arsB*, and *ACR3(2)* sequences, AsRG sequences from the isolates were compared with homologous, chromosomal sequences from related organisms deposited at the NCBI. Sequences from phylogenetic relatives were found by searching chromosomes deposited at the NCBI, and closest NCBI matches for AsRG sequences were determined using BLAST. A corresponding 16S rRNA tree was made using sequences from the isolates and their phylogenetic relatives. The sequences obtained from NCBI can be found with the following accession numbers: *Acinetobacter baumannii* strain A1 (CP010781.1), *Enterobacter cloacae* subsp. cloacae ATCC 13047 (296100371), *Pseudomonas aeruginosa* PAO1 (AE004091.2), *Enterobacter kobei* strain DSM 13645 (CP017181.1), *Escherichia coli* str. K-12 substr. MG1655 (NC_000913.3), *Enterobacter asburiae* L1 (NZ_CP007546.1), *Bacillus cereus* ATCC 10987 (AE017194.1), *Paenibacillus terrae* HPL-003 (374319880), *Bacillus thuringiensis* strain Bc601 (CP015150.1), Shewanella oneidensis MR-1 (NC_004347), *Stenotrophomonas maltophilia* K279a (AM743169.1), *Bacillus thuringiensis* strain 97–27 (CP010088.1), *Rhodoferax ferrireducens* T118 (CP000267.1), *Cyclobacterium marinum* DSM 745 (CP002955.1) Trees were constructed using MEGA7.0 [51] and constructed with the maximum likelihood algorithm using the Kimura 2 parameter model with 100 bootstrap replications. Distances between As resistance and 16S rRNA gene trees were calculated using the R environment for statistical computing [52] with the Phangorn package [53].

To further investigate evidence for HGT, the GC content of AsRG sequences was compared with reference GC content from whole genomes of related species. Reference GC content was calculated by averaging the GC content of all organisms in NCBI “Genome Groups” for the related taxon.

### Cultivation-independent 16S rRNA amplicon sequencing and analysis

Soil DNA was extracted, sequenced, and analyzed in a previous work [54] from the same sample used for isolation. Using BLAST (v. 2.2.26), a database of representative 16S rRNA gene sequences was constructed. Isolate 16S rRNA gene sequences from Sanger sequencing were used as queries against this database to find top hits and to estimate the relative abundance of our isolates in the microbial community. The top hit was determined as the hit with the highest percent identity for that isolate with a minimum percent identity of 96%, and the relative abundance of representative sequence [54] was used as the estimate of the relative abundance of each isolate.

### As transformation capabilities

The ability of the isolates to reduce arsenate or oxidize arsenite was measured using a slightly modified (described below) silver nitrate colorimetric assay as described previously [55]. 0.1 M Tris-HCl (pH 7.3) was used as a reaction buffer instead of 0.2 M, and 1.33 mM sodium arsenate or sodium arsenite was used instead of 0.67 mM. Cells were inoculated in 3 mL TSB50 and incubated at 27°C for 15 h before plating. Cells were washed with sterile reverse osmosis (RO) water to remove culture media as indicated in Simeonova *et al*. [55], and 20 μL of the washed cell suspension was incubated with 80 μL of 0.1 M Tris-HCl and 1.33 mM As in a 96-well plate for 72 h at 27°C. Two standard curves with different ratios of sodium arsenate and sodium arsenite (0:100, 10:90, 25:75, 50:50, 75:25, 90:10, 100:0) were also included alongside the cells. After a 72 h incubation, cell viability was tested. Cells were patched onto fresh TSA50 plates to test cell viability. The reaction was initiated by adding 100 μL of sterile 0.1M AgNO_3_ to each sample in the 96-well plate. After the silver nitrate reaction was initiated, plate photographs were taken, and colorimetric changes were assessed. This protocol was performed with at least two biological replicates plated in duplicate.

### Minimum inhibitory concentrations (MICs)

To determine the MICs of arsenate and arsenite as well as their growth phenotypes, isolates were inoculated from 25% glycerol stocks into 3 mL TSB50 and incubated with shaking at 200 rpm with a 12 mm amplitude at 27°C for 6 h. Inocula were added to a 96-well plate with As-containing TSB50 to make a 1% inoculum. Concentrations tested include 0, 10, 50, 100, 150, 200, 250, and 300 mM sodium arsenate and 0, 1, 3, 5, 7, 10, 14, and 20 mM sodium arsenate. Plates were shaken continuously at 288 rpm with a 3 mm amplitude in an Infinite500 plate reader (Tecan) for 72 h at 27 ± 1°C. OD_590_ was measured every 15 min. Growth experiments were repeated with at least two biological replicates for each isolate, and growth curves for further analysis were made using technical triplicates.

The R environment for statistical computing [52] was used to plot growth curves and analyze key features of growth inhibition across the range of arsenate and arsenite concentrations tested using a modified script (http://bconnelly.net/2014/04/analyzing-microbial-growth-with-r/). Using the GroFit package [56], splining was used to extract growth parameters including time to exponential growth (λ), maximum growth rate (μ), and maximum OD_590_ (A). When splining was not appropriate (e.g. curves do not have a smooth fit), parameters were estimated parametrically using either Logistic, Gompertz, or Richards models informed by their Akaike information criterion (AIC) [57]. Parameters for each isolate in TSB50 containing As were normalized to As-free controls. We used hierarchical clustering to examine similarities in growth phenotypes in As for genera with more than two representatives (n > 2). The clustering included growth parameters (λ, μ, and A) in 1 mM sodium arsenite and 10 mM sodium arsenate for each isolate. Only one As concentration was used so that MIC NA values did not impact the clustering. All R scripts are available on GitHub (https://github.com/ShadeLab/Arsenic_Growth_Analysis/tree/master/R_scripts) for future studies interested in isolate fitness in As.

## Results

### Taxonomic diversity and composition of arsenic resistant isolates

**Fig 1:**
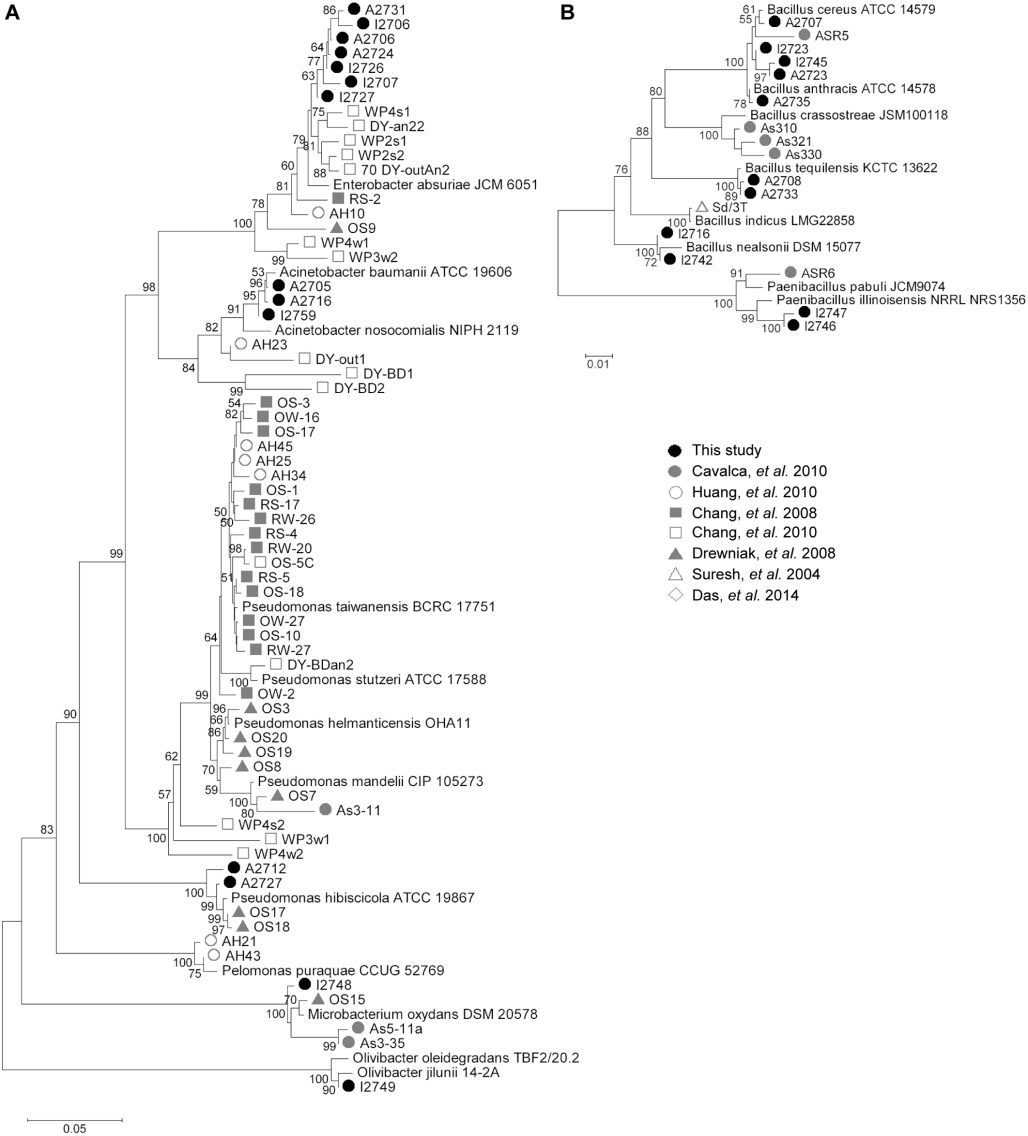
Phylogenetic tree of 16S rRNA sequences from Centralia As resistant isolates. Isolates from this study were compared with isolates from other studies that cultivated As resistant isolates from soil. (A) Actinobacteria, Proteobacteria, and Sphingobacteria. (B) Firmicutes. Scale bars indicate the percent difference in nucleotide sequence.

As resistant isolates were cultivated from soil near an active vent (S1 Table) of the Centralia coal seam fire with low As (2.58 ppm) by screening for As resistance on 10 mM sodium arsenate and 1 mM sodium arsenite. Isolates spanned seven genera, including *Acinetobacter*, *Bacillus*, *Enterobacter*, *Microbacterium*, *Olivibacter*, *Paenibacillus*, *and Pseudomonas* (Fig 1 and S4 Table). The colony morphologies of the isolates aligned with expectations given 16S rRNA gene classification (near full length sequences were obtained), and all isolates grew in 24 h at or above 39°C (S4 Table). This cultivation effort resulted in an abundance of Firmicutes (48% of isolates). To determine the relative abundances of these As resistant isolates within their larger community, we used BLAST to query isolate full-length 16S rRNA gene sequences against representative 16S rRNA gene sequences of operational taxonomic units from amplicon data (948,228 raw reads) obtained in our previous study [54]. The relative abundance of top hits for each isolate ranged from 6.23x10^-6^ to 1.59x10^-4^ (Table 1), suggesting that all As resistant isolates isolated in this study are rare members of this soil community.

**Table 1:** Relative abundance of isolate 16S rRNA gene sequences from our amplicon survey of the same soil.

Taxonomic group	Isolates	Relative abundance
*Acinetobacter*	I2759, A2705, A2716	6.23×10^-6^
*Bacillus anthrasis*	I2723, I2745, A2707, A2723, A2735	3.12×10^-6^
*Bacillus subtilis*	A2708, A2733	1.03×10^-4^
*Bacillus nealsonii*	I2716, I2742	1.59×10^-4^
*Enterobacter*	I2706, I2707, I2726, I2727, A2706, A2724, A2731	3.12×10^-5^
*Microbacterium*	I2748	3.12×10^-6^
*Paenibacillus*	I2746, I2747	3.12×10^-6^
*Pseudomonas*	A2712, A2727	9.35×10^-6^
*Olivibacter*	I2749	2.49×10^-5^

### Genetic characterization of As resistance

**Fig 2:**
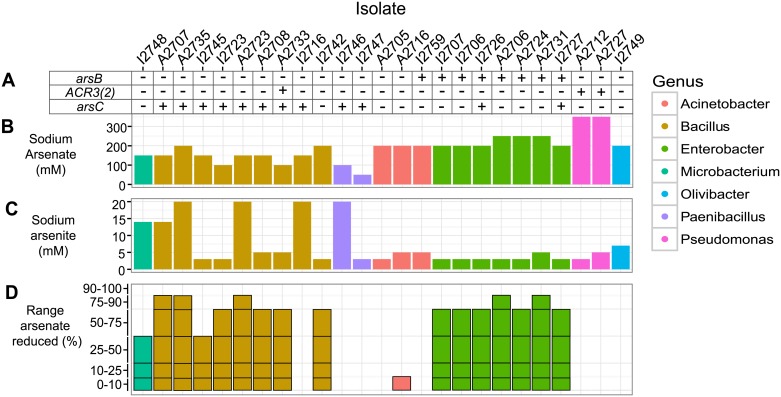
As resistance genotypes and phenotypes of isolated bacterial strains. (A) Presence of AsRG from end-point PCR are indicated (+). MICs of (B) sodium arsenate and (C) arsenite. (D) Categorical range of arsenate reduced based on standard curve of known ratios of arsenate and arsenite.

As resistance genotypes of the isolates were characterized using endpoint polymerase chain reaction (PCR) with a collection of published primers (S2 Table) specific for genes encoding resistance via diverse mechanisms, including arsenate reduction, arsenite oxidation, methylation, and arsenite efflux (Fig 2A). After endpoint PCR, all amplicons were sequenced to confirm their identities. Eight isolates (32%) had the gene encoding the arsenite efflux pump, *arsB*. The majority of *arsB*-positive isolates belonged to the genus *Enterobacter* with the exception of one *Acinetobacter* isolate. Three isolates (12%) had the gene encoding arsenite efflux pump, *ACR3(2)*. Twelve isolates (48%) had the arsenate reductase gene, *arsC*. We did not find evidence for genes encoding other resistance mechanisms including dissimilatory arsenate reductase (*arrA*), arsenite oxidase (*aioA*), arsenite efflux pump (*ACR3(1)*), or arsenite methyltransferase (*arsM*) in the isolate collection. Thus, only genes related to arsenate reduction and arsenite extrusion were detected among these Centralia isolates using prominent primer sets. Notably, five isolates (20%) did not test positive for any AsRGs tested using published primers, suggesting sequence diversity of tested genes that are not captured with these primer sets, undescribed resistance genes, or resistance through general stress responses.

### As transformation

We determined the abilities of isolates to transform arsenate and arsenite using a published semiquantitative measure of percent As transformation without growth media [55]. No isolates oxidized arsenite in this assay (data not shown). However, we observed a wide range of capabilities for arsenate reduction that generally corresponded to isolate taxonomy (Fig 2D). All isolates belonging to the genus *Enterobacter* had transformation capabilities at or above 50%. Isolates belonging to *Bacillus* had varied arsenate reduction capabilities ranging from 0–90%. The *Microbacterium* isolate (I2748) reduced 10–25% of arsenate in solution, and *Acinetobacter* isolates reduced 0–10% of arsenate. While nine isolates (36%) reduced arsenate *in vitro* and tested positive for *arsC*, there were discrepancies between the *in vitro* and genetic data. Isolates belonging to genera *Olivibacter*, *Paenibacillus*, and *Pseudomonas* did not reduce arsenate in this assay (Fig 2D). An additional three isolates (12%) tested positive for *arsC* but did not reduce arsenate in this assay. It is possible that *arsC* is nonfunctional in these bacterial strains, not active in these conditions, or that arsenate reduction occurred but was below the limit of detection of this assay. Additionally, eight isolates (32%) reduced arsenate in this assay but did not test positive for the genes encoding arsenate reductases (*arsC* or *arrA*). These isolates may contain less characterized arsenate reductase genes [58].

### Incongruent phylogenies of As resistance and 16S rRNA genes

**Fig 3:**
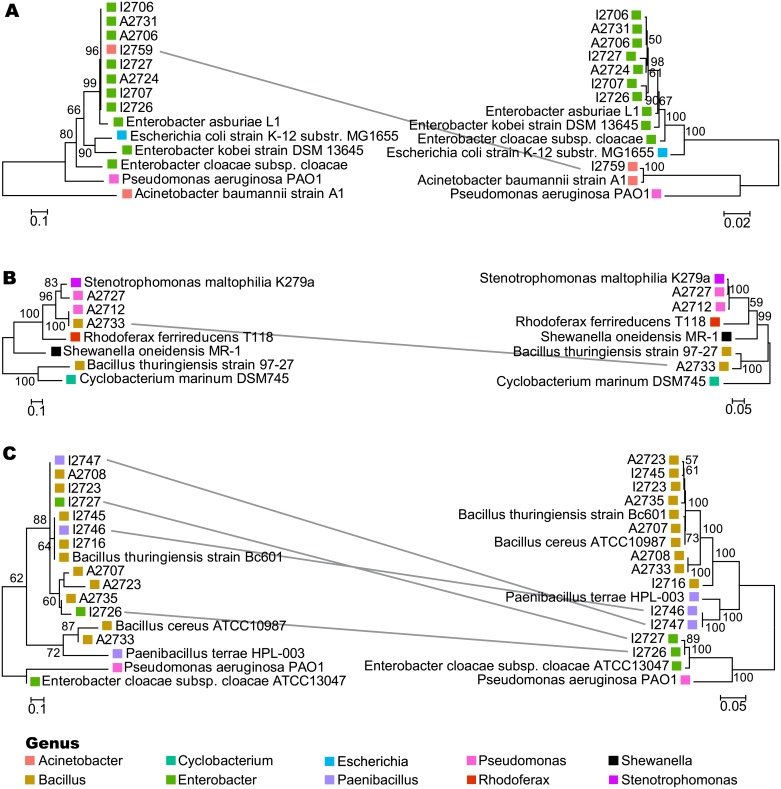
Comparison of AsRG sequences and 16S rRNA gene sequences from As resistant isolates. Maximum likelihood trees for AsRGs (left panel) (A) *arsB*, (B) *ACR3(2)*, and (C) *arsC* are shown alongside trees of corresponding 16S rRNA genes (right panel). Incongruence is highlighted with grey lines between the two trees. Scale bars indicate the percent difference in nucleotide sequence. Bootstrap values greater than 50% are indicated at the corresponding node, and boxes are colored based on isolate genus.

Maximum likelihood trees of detected AsRGs were compared with their corresponding 16S rRNA gene trees, and there was incongruence in all instances (Fig 3). All *arsB* sequences were related to *Enterobacter*, including those from an *Acinetobacter* isolate (Fig 3A). Three isolates spanning two genera (*Pseudomonas*, *Bacillus*) tested positive for *ACR3(2)*, and all had high sequence homology to *Stenotrophomonas*-derived *ACR3(2)* (Fig 3B). Comparing the *arsC* and 16S rRNA phylogenetic trees revealed several inconsistencies between gene sequence and phylogeny (Fig 3C). Twelve isolates spanning three genera (*Bacillus*, *Paenibacillus*, and *Enterobacter*) had high sequence homology to *Bacillus*-derived *arsC*, suggesting HGT. Closest NCBI BLAST hit and GC content for each AsRG and corresponding taxa further suggested incongruence (S5 Table). Collectively, these data suggest past, and potential future, movement of these AsRGs via HGT.

### MICs and growth phenotypes in As

In parallel to characterization of genetic mechanisms of As resistance, we determined the MICs of arsenate and arsenite for each isolate (Fig 2B and 2C). MIC phenotypes ranged from 50 mM to >300 mM for sodium arsenate and from 3 to 20 mM for sodium arsenite. Both *Pseudomonas* isolates could withstand >300 mM sodium arsenate, which is typical for previously reported pseudomonads resistant to As [18,59]. High sodium arsenate resistance (>200 mM) [60] was observed in 20% of the isolates. High sodium arsenite resistance (>15 mM) [18] was observed in 16% of the isolates, all of which belong to phylum Firmicutes.

**Fig 4:**
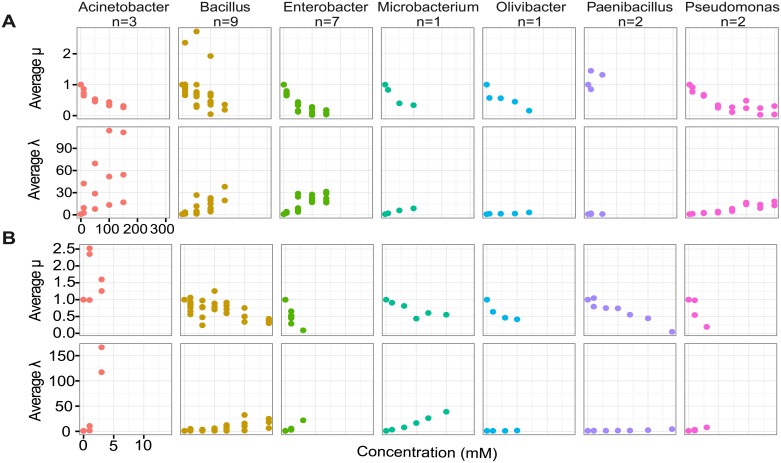
Growth phenotypes of isolates in increasing concentrations of As. Lag time (λ) and maximum growth rate (μ) of isolates in TSB50 with increasing concentrations of (A) arsenate and (B) arsenite normalized to growth without As.

We also analyzed growth phenotypes (lag time, maximum growth rate, and maximum OD_590_) in As, and our results highlight a nuanced relationship between growth in As and taxonomy that was more informative than the observed MIC data alone (Fig 4, S1 and S2 Figs). Limited conclusions can be made about *Paenibacillus*, *Microbacteriun*, *Olivibacter*, and *Pseudomonas* isolates due to the small sample size (n ≤ 2) of these genera. Maximum growth rate (μ) and maximum OD_590_ (A) showed similar patterns in each isolate, so we only report μ here and provide A in supporting materials (S2 Fig). In general, relative growth phenotypes were similar between arsenate and arsenite. Firmicutes isolates maintained basal growth rates in the presence of As. Here we offer a qualitative description of the isolates’ growth phenotypes in As. More work will be needed to understand how general these growth phenotypes may be within lineages. While *Paenibacillus* isolates had the lowest MICs, they showed the least overall growth phenotype change in As. *Bacillus* isolates, however, exhibited larger increases in lag time (**λ**) as compared with *Paenibacillus* isolates. Conversely, the *Olivibacter* isolate showed an increase in lag time along with reductions in growth rate. Again, because there was only one *Olivibacter* isolate, we cannot know how general its growth trends in As are. Members of *Enterobacter* had reductions in growth rate as well as increased lag time with increasing As concentrations despite their high MICs. Hierarchical clustering of growth phenotypes in genera with more than two isolates revealed clustering based on taxonomy rather than genotype or MIC (S3 Fig). Despite variability in **λ** in *Acinetobacter* isolates, they clustered apart from *Enterobacter* and *Bacillus* and had comparably higher values. Similarly, *Bacillus* strains clustered together despite variability in μ observed within genus. Again, because we have limited representatives of *Paenibacillus*, *Pseudomonas* and *Microbacterium*, future studies should investigate the generality of their growth phenotypes in arsenic. These results suggest that, aside from the concentration of As exposure, growth changes in lag time, rate, and maximum OD may impact an isolate’s survival outcomes *in situ*. More work is needed to determine if collective growth phenotype changes among As resistant isolates within a soil community may be in part predicted by taxonomy and by occurrence of HGT.

## Discussion

Our results from characterizing this modest isolate collection of As resistant soil bacteria expose two considerations regarding the microbial community ecology of As exposure. First, our data show that members of the rare biosphere harbor AsRGs that appear to be transferred via HGT in the past and therefore could have potential for transfer in the future. Second, our results suggest that nuanced growth phenotypes in As may be predictable by the taxonomic identity of the microorganism that has not been described previously. This has implications for understanding a microbial community’s response to As, as it suggests there are differential growth responses, and therefore different competitive abilities, of resistant taxa. Thus, while the distribution and transfer of AsRGs in the microbial community have implications for filtering of community members given As exposure, knowledge of As growth phenotypes could be used to predict the compositional outcome (re-structuring) of an As-exposed community; however, more work examining consistency of growth phenotypes in As within and among lineages would inform the feasibility of such forecasting.

In this study, we described a collection of 25 aerobic As resistant bacterial strains isolated from soils of active vent from an underground coal seam fire in Centralia, PA, a unique terrestrial environment. We subsequently determined that, despite the fire activity at this particular site, the soil had relatively low As concentrations at the time of soil collection (2.58 ppm). This is not surprising, given that 1) the fire is dynamic and past As concentrations at the vent may have been higher given the natural occurrence of As as a byproduct of coal combustion [32,34] and 2) the widespread observation of microbial As resistance from soils that have generally low contamination [41,60–63]. While our isolation resulted in an abundance of Firmicutes, this is not surprising since members of phylum Firmicutes have been shown to be resistant to As previously with varied MICs [60,64]. Additionally, we acknowledge cultivation bias and that freezing soil prior to cultivation may have influenced our ability to resuscitate some strains [65]. Accordingly, all 25 isolates were rare within their soil microbial community (Table 1). Previous studies have shown that cultivation from soil can isolate rare community members [30], but this is the first specific documentation of enrichment of As resistant bacteria from the rare biosphere. This is relevant to the Centralia community because soil As concentrations may increase due to coal combustion [32,34]. While we cannot determine the response of the general community to additional As deposition, our results suggest that members of the rare biosphere are capable of surviving As stress and have potential to transfer resistance genes.

We also found that growth phenotypes in As provided richer context for tolerance than MICs. Our results are consistent with previous reports that Proteobacteria often have high MICs (Fig 2B) [5,19]; however, when simultaneously analyzing reductions in growth with As, our results show distinct growth strategies among lineages, in both arsenate and arsenite (Fig 4 and S3 Fig). While other reports have examined growth reduction in the presence of As to find suitable strains for bioremediation [17,62,63,66,67], a suite of growth parameters are not typically investigated. Our full characterization of growth in increasing concentrations of As showed a modest relationship between growth phenotype and taxonomy and highlights discrepancies between fitness in As and MIC. This taxonomic delineation of growth phenotypes may be attributed to lineage-distinct mechanisms of As tolerance; however, limited conclusions can be made for genera with small sample sizes (*Paenibacillus*, *Microbacteriun*, *Olivibacter*, and *Pseudomonas)*. Jobby and colleagues [10] found an increased lag time with As addition in an *Enterobacter* isolate from Navi Mumbai, which is similar to the lag times observed of *Enterobacter* isolates from Centralia, PA. This further implicates taxonomy as an important factor in an organism’s tolerance to As in liquid culture. Accounting for tolerance mechanisms may explain some of the discrepancies between MIC and As resistance genotype [41] and between MIC and isolate abundance in contaminated sites [64]. Valverde and colleagues [64] observed an increase in Firmicutes with increasing As concentrations despite their lower MICs *in vitro*. Our findings suggest that As resistant Firmicutes, in general, had modest changes in growth phenotypes in As. Generally, this result questions the precision of MICs in predicting the success of a microorganism in the presence of As. While this report is descriptive and not an exhaustive look at the relationship between growth phenotype in As and taxonomy, consideration of both growth phenotype and taxonomy may offer additional predictive value and future studies should further examine growth phenotypes in As.

Microbial arsenate reduction and the transfer of associated functional genes are important environmental health concerns because these processes increase the mobility of environmental As [4]. Incongruence between the phylogenetic alignment of *arsC*, *arsB*, and *ACR3(2)* and the 16S rRNA gene within this isolate collection suggests horizontal transfer of AsRGs (Fig 3), despite low As and therefore low direct-selection pressure at this site. Determining the genetic environment of these AsRGs (chromosomal location or plasmid-borne) through whole genome sequencing would further determine whether these genes were horizontally transferred and provide insights into mechanisms of transfer. These results further emphasize the potential HGT seen of genes encoding arsenite efflux pumps and arsenate reductase seen previously [6,19]. Specifically, HGT of the gene encoding arsenite efflux pump (*arsB*) has been seen in environments with low As concentrations [19]. Notably, these data indicate potential HGT from multiple species, suggesting community-level contributions to As resistance rather than a limited source of resistance genes. Investigating interactions among community members in the context of As contamination may provide insights into the sources and sinks underlying the movement of resistance genes.

Finally, we observe multiple discrepancies between genetic and functional assays when characterizing the isolates’ As resistance. Despite using twelve published and commonly used primer sets to screen for AsRGs, three isolates with relatively high MICs did not test positive for any AsRGs screened in this study, highlighting a caveat of using primers for detection [5,41]. We also observe inconsistencies between genetic results and arsenate transformation capabilities, suggesting divergent gene sequences, presence of untested AsRGs (including the possibility of novel genes [20]), or general stress responses. A wider breadth of AsRG diversity is likely to be captured using complementary cultivation-independent methods.

Our focus on growth phenotypes in As revealed a relationship with taxonomy that has not been described previously. Additionally, our data show that rare community members can exhibit As resistance and contain AsRGs. These observations have implications not only for As tolerance but also for mechanisms supporting general microbial community robustness to As stress.

## Supporting information

S1 Fig Average OD590 over 72 h in TSB50 with in increasing concentrations of arsenate (A) or arsenite (B). Grey ribbon represents 95% confidence intervals from three replicates. Note the difference in color scales for A and B.
(PDF)

S2 Fig Growth phenotypes in TSB50 with increasing concentrations of arsenate and arsenite normalized to growth in TSB50 without arsenic. Points are averages from three technical replicates, and error bars show standard deviation. Note the different scale for λ in arsenite for isolatesA2705, A2716, and I2759.
(PDF)

S3 Fig Dendrogram of isolate growth phenotypes in As. Only isolates belonging to genera with n > 2 are included. Growth parameters (λ, μ, A) in 1 mM sodium arsenite and 10 mM sodium arsenate were normalized to those with no As and used for clustering. Color indicates isolate genus.
(EPS)

S1 Table Phenotypes of arsenic resistant isolates. (PDF)

S2 Table Degenerate primers used for end point PCR. (PDF)

S3 Table Isolates with short arsC sequences (<200 bp). (PDF)

S4 Table Phenotypes of arsenic resistant isolates. (PDF)

S5 Table Comparison of As resistance gene sequences with NCBI references. Top BLAST hit is recorded for each As resistance gene contig. %GC of the contig is also listed alongside average %GC of reference genomes (with standard deviation shown) of the same taxonomy. Sequences were accessed from NCBI on January 12, 2017.
(PDF)
