# Supplementary figures and images for: Taxonomically-linked growth phenotypes during arsenic stress among arsenic resistant bacteria isolated from soils overlying the Centralia coal seam fire
Source: PLoS One. 2018 Jan 25;13(1):e0191893. doi: 10.1371/journal.pone.0191893 (PMC5785013; doi:10.1371/journal.pone.0191893)

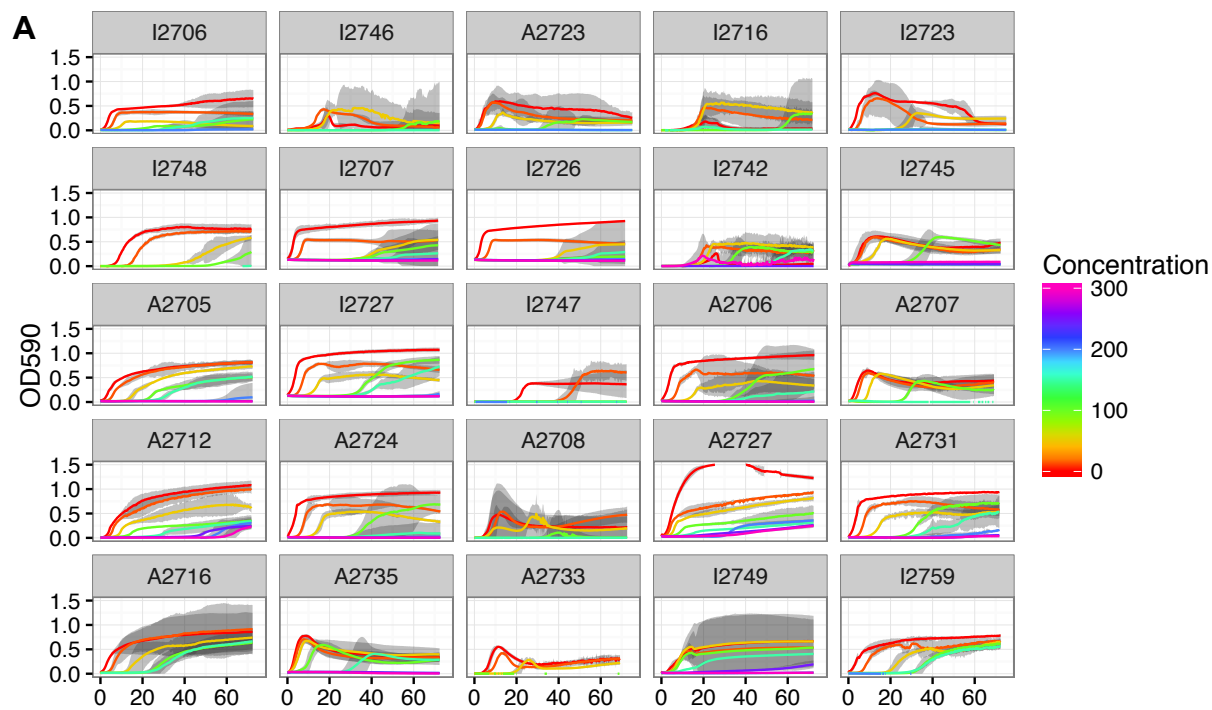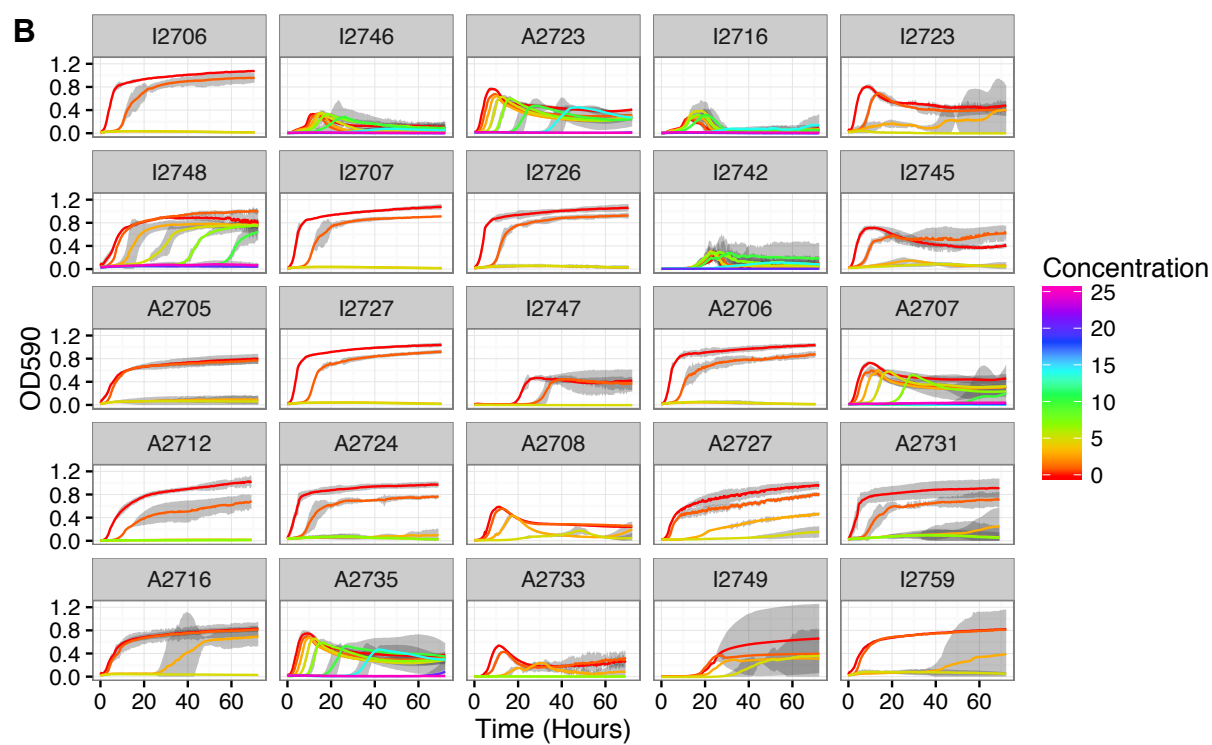

Supplement: S1 Fig — Grey ribbon represents 95% confidence intervals from three replicates. Note the difference in color scales for A and B. (PDF) [file pone.0191893.s001.pdf]

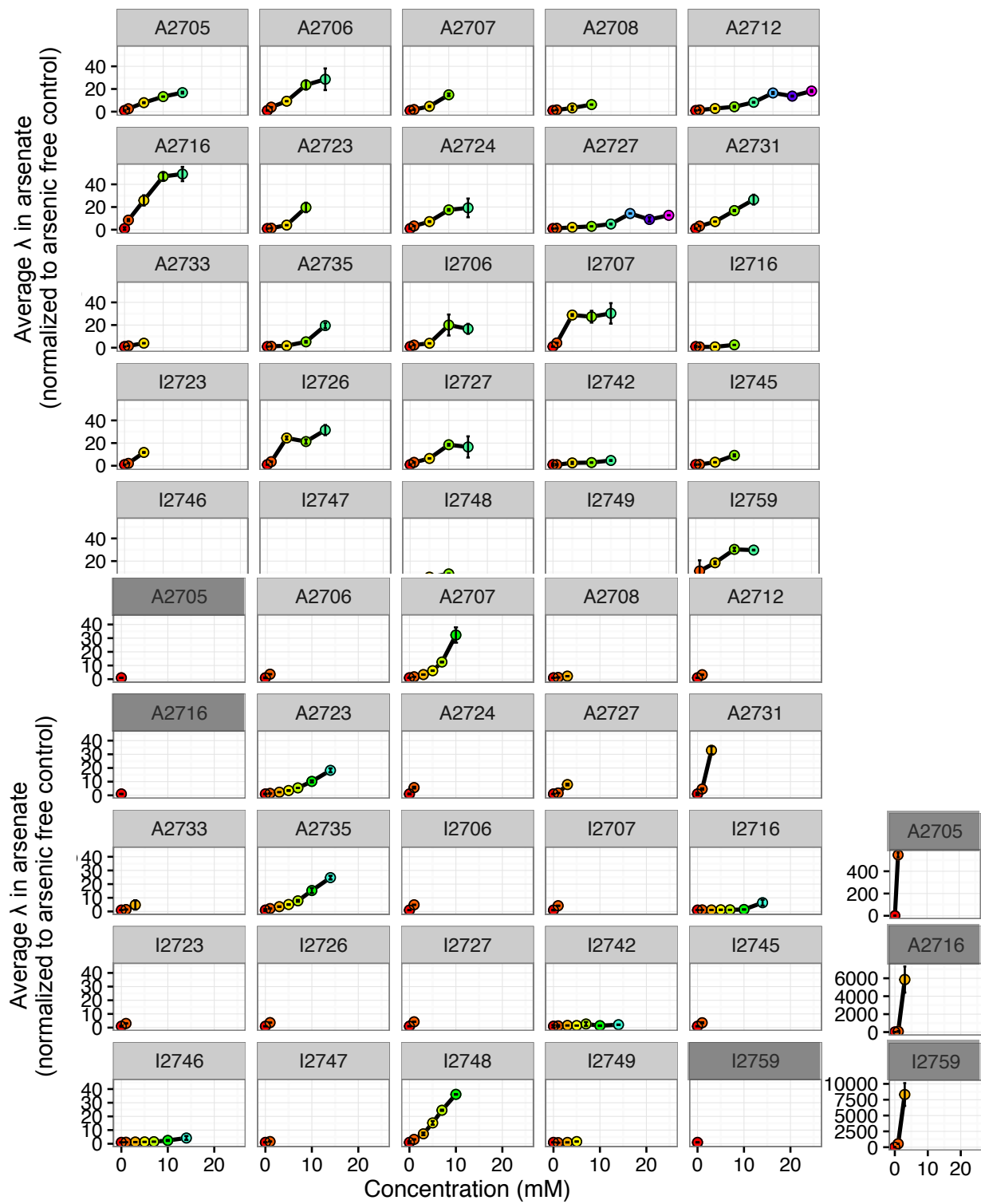

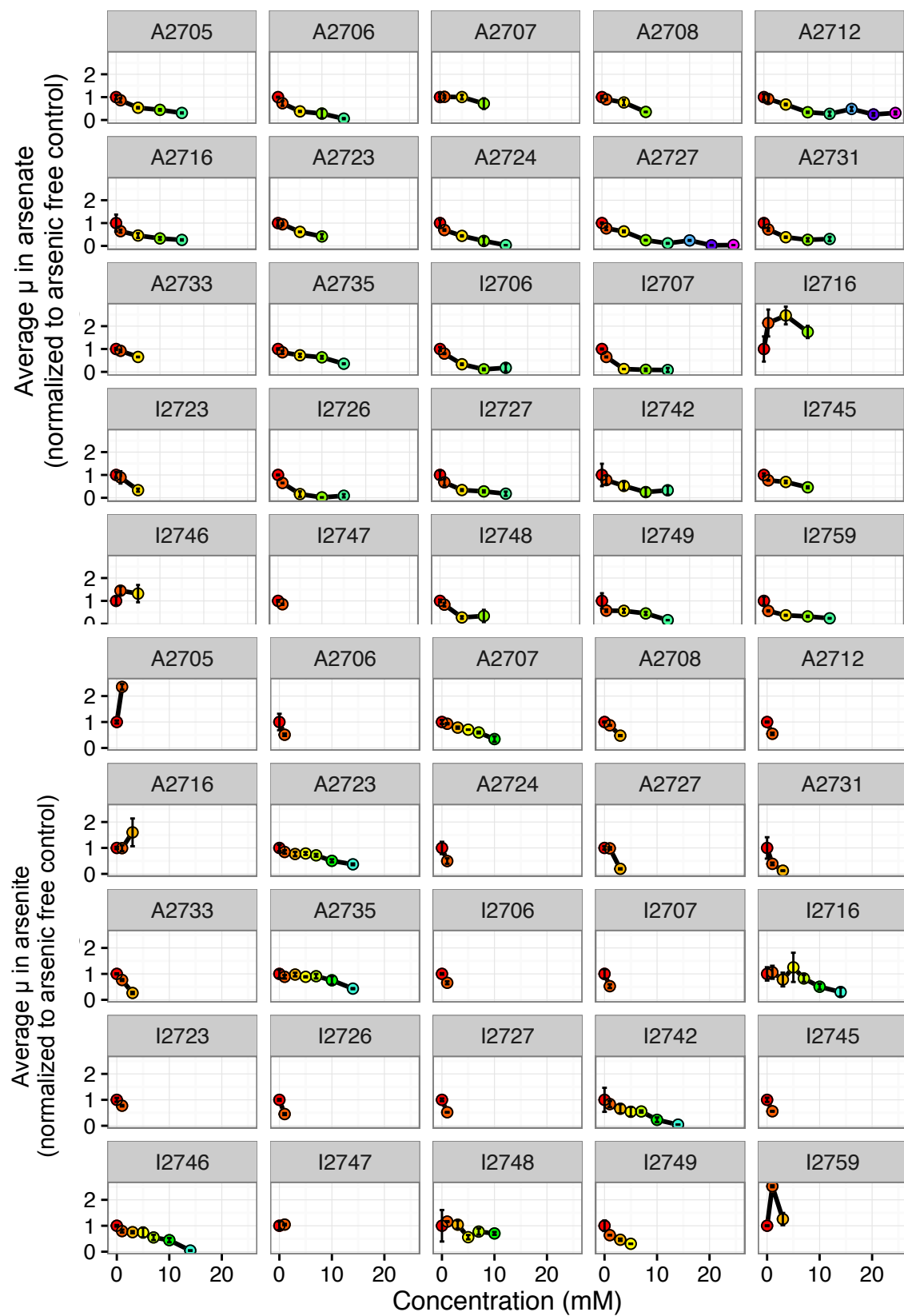

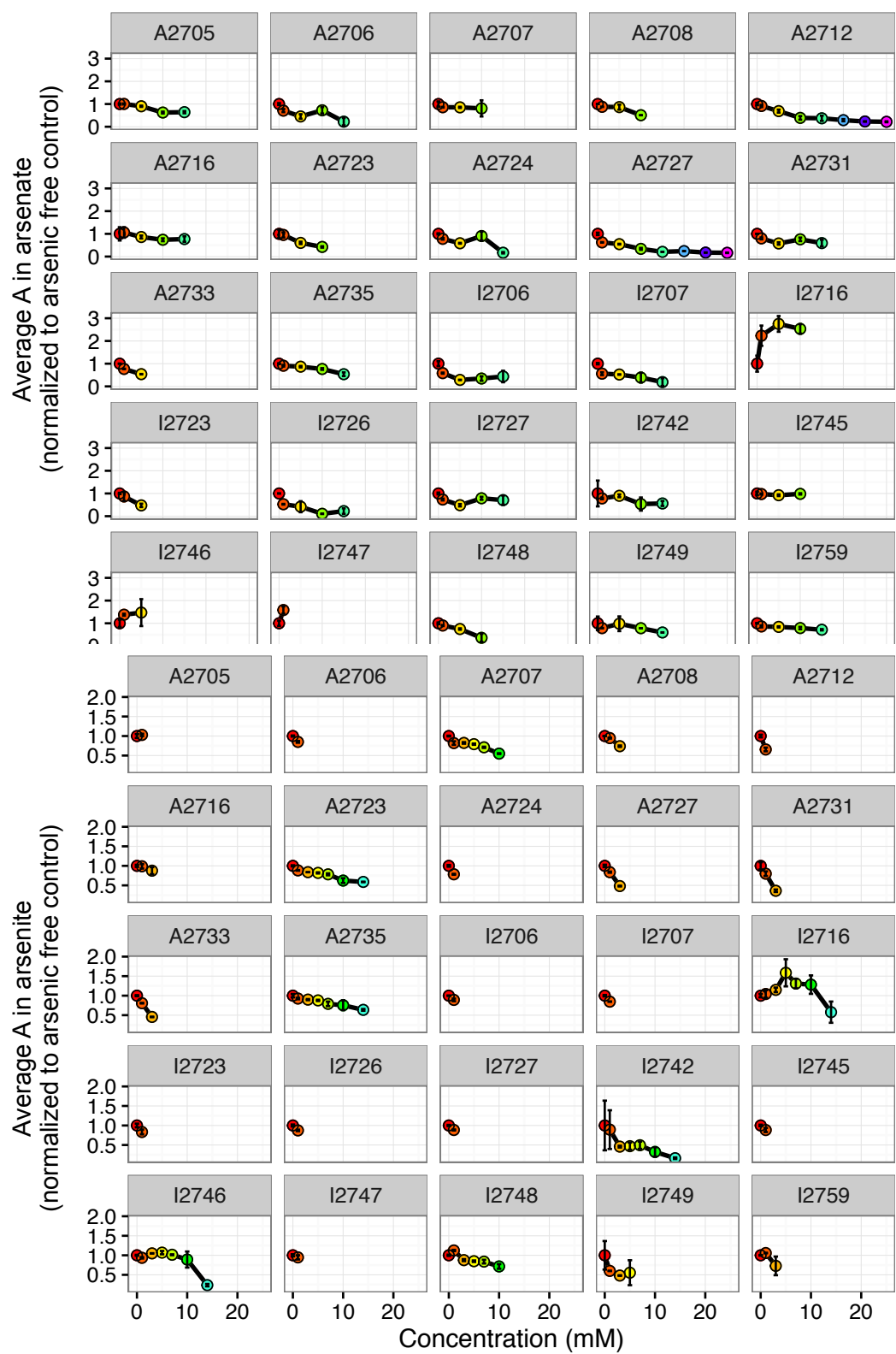

Supplement: S2 Fig — Points are averages from three technical replicates, and error bars show standard deviation. Note the different scale for λ in arsenite for isolatesA2705, A2716, and I2759. (PDF) [file pone.0191893.s002.pdf]
