# Supplementary material for: Taxonomically-linked growth phenotypes during arsenic stress among arsenic resistant bacteria isolated from soils overlying the Centralia coal seam fire
Source: PLoS One. 2018 Jan 25;13(1):e0191893. doi: 10.1371/journal.pone.0191893 (PMC5785013; doi:10.1371/journal.pone.0191893)
Supplement: S1 Table — (PDF) [file pone.0191893.s004.pdf]

**Air temperature (°C)**	**Soil temperature (°C)**	**Organic matter (360°C)**	**Organic matter (500°C)**	**NO_3^- (ppm)**	**NO_4^+ (ppm)**	**pH**	**S (ppm)**	**K (ppm)**	**Ca (ppm)**	**Mg (ppm)**	**Fe (ppm)**	**As (ppm)**
13.3	57.4	3.1	7.1	4.6	1.7	8	28	37	2545	114	67.1	2.58
