# Supplementary material for: Taxonomically-linked growth phenotypes during arsenic stress among arsenic resistant bacteria isolated from soils overlying the Centralia coal seam fire
Source: PLoS One. 2018 Jan 25;13(1):e0191893. doi: 10.1371/journal.pone.0191893 (PMC5785013; doi:10.1371/journal.pone.0191893)
Supplement: S2 Table — (PDF) [file pone.0191893.s005.pdf]

Gene	Primer Sequence (5'-3')	Name	Source
16S	AGAGTTTGATCCTGGCTCAG	Uni-27F	Weisburg *et al*., 1991
16S	GGTTACCTTGTTACGACTT	Uni-1492R	Weisburg *et al*., 1991
16S	GTGCCAGCMGCCGCGGTAA	U515F	Baker *et al*., 2003
arsB	GGTGTGGAACATCGTCTGGAAYGCNAC	darsB1F	Achour *et al*., 2007
arsB	CAGGCCGTACACCACCAGRTACATNCC	darsB1R	Achour *et al*., 2007
ACR3(1)	GCCATCGGCCTGATCGTNATGATGTAYCC	dacr1F	Achour *et al*., 2007
ACR3(1)	CGGCG ATGGCCAGCTCYAAYTTYTT	dacr1R	Achour *et al*., 2007
ACR3(2)	TGA TCTGGGTCATGATCTTCCCVATGMTGVT	dacr5F	Achour *et al*., 2007
ACR3(2)	CGGCCACG GCCAGYTCRAARAARTT	dacr4R	Achour *et al*., 2007
arsC	TCGCGTAATACGCTGGAGAT	amlt-42-f	Sun *et al*., 2004
arsC	ACTTTCTCGCCGTCTTCCTT	amlt-376-r	Sun *et al*., 2004
arsC	TCACGCAATACCCTTGAAATGATC	smrc-42-f	Sun *et al*., 2004
arsC	ACCTTTTCACCGTCCTCTTTCGT	smrc-376-r	Sun *et al*., 2004
arsC	AGCCAAATGGCAGAAGC	P52F	Cavalca, *et al*., 2010
arsC	GCTGGRTCRTCAAATCCCCA	P323R	Cavalca, *et al*., 2010
arrA	CGAAGTTCGTCCCGATHACNTGG	AS1F	Song *et al*., 2009
arrA	GGGGTGCGGTCYTTNARYTC	AS1R	Song *et al*., 2009
arrA	GTCCCNATBASNTGGGANRARGCNMT	AS2F	Song *et al*., 2009
arrA	ATANGCCCARTGNCCYTGNG	AS2R	Song *et al*., 2009
aioA	CCACTTCTGCATCGTGGGNTGYGGNTA	aoxBM1-2F	Quemeneur *et al*., 2008
aioA	TGTCGTTGCCCCAGATGADNCCYTTYTC	aoxBM3-2R	Quemeneur *et al*., 2008
arsM	TCYCTCGGCTGCGGCAAYCCVAC	arsMF1	Jia *et al*., 2013
arsM	GTGCTCGAYCTSGGCWCCGGC	arsMF2	Jia *et al*., 2013
arsM	GGCATCGACGTGCTKCTBTCSGC	arsMF3	Jia *et al*., 2013
arsM	AGGTTGATGACRCAGTTWGAGAT	arsMR1	Jia *et al*., 2013
arsM	CGWCCGCCWGGCTTWAGYACCCG	arsMR2	Jia *et al*., 2013
arsM	GCGCCGGCRAWGCAGCCWACCCA	arsMR3	Jia *et al*., 2013
