# Supplementary material for: Taxonomically-linked growth phenotypes during arsenic stress among arsenic resistant bacteria isolated from soils overlying the Centralia coal seam fire
Source: PLoS One. 2018 Jan 25;13(1):e0191893. doi: 10.1371/journal.pone.0191893 (PMC5785013; doi:10.1371/journal.pone.0191893)
Supplement: S3 Table — (PDF) [file pone.0191893.s006.pdf]

Isolate	*arsC* sequence
A2707	cgatgctgatttagtctgttacgcttttgtggccatgcggatgctgtttgtccgtctactccgccgcatgtgaatcgagttcactggggatttgacgacccagcaa
A2723	gctggatttagtctgtnacnctttgtggtcacgcagatgctgtctgtccnncaacacctccgcacgtgaancgagttcactggggatttgacgacccagcaa
A2733	caatgaanatcnggatggtttgattccgttatgatcgatgtctacttcgtttcattgctttaattgcnttcggattcactccgtgtgcctcnatacccgcagaaantac
A2735	cgatgctgatttagtctgttacgctttgtggtcacgcagatgctgtctgtccnncaacacctcctcacgtgaancgagttcactggggatttgacgacccagcaaa
