# Supplementary material for: Taxonomically-linked growth phenotypes during arsenic stress among arsenic resistant bacteria isolated from soils overlying the Centralia coal seam fire
Source: PLoS One. 2018 Jan 25;13(1):e0191893. doi: 10.1371/journal.pone.0191893 (PMC5785013; doi:10.1371/journal.pone.0191893)
Supplement: S4 Table — (PDF) [file pone.0191893.s007.pdf]

Isolate	Closest 16S rRNA gene sequence described (% similarity)	Colony Morphology	Temperature Maximum (°C)	Length (µm)	Width (µm)
I2706	*Enterobacter absuriae* JM 6051 (99.43%)	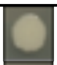	44.3	1.43	1.21
I2707	*Enterobacter absuriae* JM 6051 (99.35%)	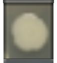	44.3	1.34	1.16
I2716	*Bacillus nealsonii* DSM 150-7577 (99.49%)	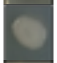	44.3	4.63	1.16
I2723	*Bacillus anthrasis* ATCC 14578 (100%)	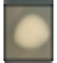	44.3	4.46	1.36
I2726	*Enterobacter absuriae* JM 6051 (99.5%)	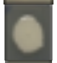	44.3	2.37	1.45
I2727	*Enterobacter absuriae* JM 6051 (99.56%)	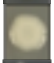	44.3	2.89	1.05
I2742	*Bacillus nealsonii* DSM 150-7577 (99.49%)	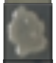	44.3	2.60	0.85
I2745	*Bacillus anthrasis* ATCC 14578 (99.86%)	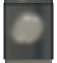	44.3	4.11	0.90
I2746	*Paenibacillus xylanilytius* XIL14 (98.63%)	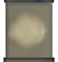	44.3	1.68	1.46
I2747	*Paenibacillus xylanilytius* XIL14 (98.58%)	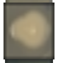	39.7	3.96	1.12
I2748	*Mirobaterium paraoxydans* F36 (99.85%)	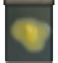	47.7	1.19	1.14
I2749	*Olivibater oleidegrans* TBF2/20.2 (99.42%)	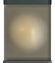	44.3	1.58	1.23
I2759	*Acinetobacter baumanii* ATCC 19606 (99.78%)	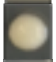	44.3	1.20	1.16
A2705	*Acinetobacter baumanii* ATCC 19606 (99.64%)	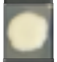	44.3	1.25	1.19
A2706	*Enterobacter absuriae* JM 6051 (99.50%)	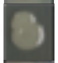	44.3	2.07	1.10
A2707	*Bacillus anthrasis* ATCC 14578 (100%)	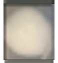	44.3	3.83	0.91
A2708	*Bacillus subtilis subsp. inoquosorum* KT 13429 (99.93%)	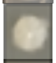	52.0	3.26	0.90
A2712	*Pseudomonas hibisicola* ATCC 19867 (99.36%)	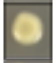	39.7	2.01	1.01
A2716	*Acinetobacter baumanii* ATCC 19606 (99.78%)	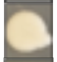	44.3	1.12	0.96
A2723	*Bacillus anthrasis* ATCC 14578 (99.73%)	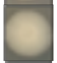	44.3	3.20	1.64
A2724	*Enterobacter absuriae* JM 6051 (99.57%)	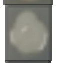	44.3	1.19	1.09
A2727	*Pseudomonas geniculata* ATCC 19374 (99.78%)	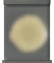	39.7	1.23	0.79
A2731	*Enterobacter absuriae* JM 6051 (99.49%)	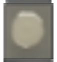	44.3	1.18	1.15
A2733	*Bacillus subtilis subsp. inoquosorum* KT 13429 (99.93%)	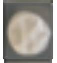	52.0	3.61	0.91
A2735	*Bacillus anthrasis* ATCC 14578 (99.85%)	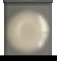	44.3	4.30	2.04
