# Supplementary material for: Taxonomically-linked growth phenotypes during arsenic stress among arsenic resistant bacteria isolated from soils overlying the Centralia coal seam fire
Source: PLoS One. 2018 Jan 25;13(1):e0191893. doi: 10.1371/journal.pone.0191893 (PMC5785013; doi:10.1371/journal.pone.0191893)
Supplement: S5 Table — Top BLAST hit is recorded for each As resistance gene contig. %GC of the contig is also listed alongside average %GC of reference genomes (with standard deviation shown) of the same taxonomy. Sequences were accessed from NCBI on January 12, 2017. (PDF) [file pone.0191893.s008.pdf]

Isolate	Genus	Gene	Closest NCBI match (% similarity)	%GC gene	%GC reference genomes
I2706	*Enterobacter*	*arsB*	*Enterobacter cloacae* isolate MBRL1077 (97%)	59.62	55 ± 0.37
I2707	*Enterobacter*	*arsB*	*Enterobacter cloacae* isolate MBRL1077 (97%)	59.42	55 ± 0.37
I2726	*Enterobacter*	*arsB*	*Enterobacter cloacae* isolate MBRL1077 (97%)	59.51	55 ± 0.37
I2727	*Enterobacter*	*arsB*	*Enterobacter cloacae* isolate MBRL1077 (97%)	59.83	55 ± 0.37
I2759	*Acinetobacter*	*arsB*	*Enterobacter cloacae* isolate MBRL1077 (97%)	59.69	39.03 ± 0.11
A2706	*Enterobacter*	*arsB*	*Enterobacter cloacae* isolate MBRL1077 (97%)	59.14	55 ± 0.37
A2724	*Enterobacter*	*arsB*	*Enterobacter cloacae* isolate MBRL1077 (97%)	59.78	55 ± 0.37
A2731	*Enterobacter*	*arsB*	*Enterobacter cloacae* isolate MBRL1077 (97%)	60	55 ± 0.37
A2712	*Pseudomonas*	*ACR3(2)*	*Stenotrophomonas maltophilia* strain ISMMS2 (84%)	62.96	66.54 ± 0.25
A2727	*Pseudomonas*	*ACR3(2)*	*Stenotrophomonas maltophilia* strain ISMMS2 (95%)	64.69	66.54 ± 0.25
A2733	*Bacillus cereus*	*ACR3(2)*	*Stenotrophomonas maltophilia* D457 (83%)	64.83	43.89 ± 0.71
I2716	*Bacillus nealsonii*	*arsC*	*Bacillus cereus* ATCC 14579 (95%)	39.5	35.1
I2723	*Bacillus cereus*	*arsC*	*Bacillus cereus* ATCC 10987 (96%)	41.2	35.26 ± 0.18
I2726	*Enterobacter*	*arsC*	*Bacillus cereus* F837/76 (99%)	40.97	55 ± 0.37
I2727	*Enterobacter*	*arsC*	*Bacillus cereus* ATCC 10987 (96%)	40.89	55 ± 0.37
I2745	*Bacillus cereus*	*arsC*	*Bacillus thuringiensis* strain KNU-07 (98%)	37.67	35.26 ± 0.18
I2746	*Paenibacillus*	*arsC*	*Bacillus cereus* strain A1 (98%)	37.8	50.9 ± 0.14
I2747	*Paenibacillus*	*arsC*	*Bacillus* sp. ABP14 (95%)	39.29	50.9 ± 0.14
A2707	*Bacillus cereus*	*arsC*	*Bacillus cereus* D17 (92%)	43.28	35.26 ± 0.18
A2708	*Bacillus subtilis*	*arsC*	*Bacillus anthracis* strain Tyrol 4675 (95%)	40.38	43.89 ± 0.71
A2723	*Bacillus cereus*	*arsC*	*Bacillus* sp. CH19 (86%)	42.79	35.26 ± 0.18
A2733	*Bacillus subtilis*	*arsC*	*Bacillus cereus* strain FORC_024 (88%)	40.1	43.89 ± 0.71
A2735	*Bacillus cereus*	*arsC*	*Bacillus* sp. CH19 (88%)	42.29	35.26 ± 0.18
